# Expanded program of immunization coverage and associated factors among children age 12 – 23 months in Arba Minch town and Zuria District, Southern Ethiopia, 2013

**DOI:** 10.1186/1471-2458-14-464

**Published:** 2014-05-17

**Authors:** Worku Animaw, Wondimagegn Taye, Behailu Merdekios, Marilign Tilahun, Gistane Ayele

**Affiliations:** 1 Bahir Dar University, College of Medicine and Health Sciences, PO Box 79, Bahir Dar, Ethiopia; 2 UNDP, Arba Minch, Ethiopia; 3 Arba Minch University, College of Medicine and Health Sciences, PO BOX 21, Arba Minch, Ethiopia

**Keywords:** Child immunization, Immunization coverage, Ethiopia

## Abstract

**Background:**

Immunization averts an estimated 2 to 3 million deaths every year globally. In Ethiopia only quarter of children are fully immunized; the rest are remained at risk for vaccine-preventable mortality. To increase the immunization, its coverage and predictors has to be identified. This study has measured immunization coverage and identified the predictors.

**Methods:**

Cross-sectional community based study has been conducted within 630 age 12–23 months children in 15 districts of Arba Minch town and Arba Minch Zuria district, Southern Ethiopia in March 2013. Census was done to identify eligible children. The 2005 world health organization expanded program of immunization cluster sampling method has been used. Data were collected using semi-structured pretested Amharic version questionnaire by interviewing index children’s mothers/caretakers, copying from vaccine card and observing BCG vaccine scar. Data were processed using SPSS version 16. Associations between dependent and independent variables has been assessed and presented using three consecutive logistic regression models.

**Result:**

Nearly three fourth (73.2%) of children in Arba Minch Town and Arba Minch Zuria district were fully immunized. The rest 20.3% were partially immunized and 6.5% received no vaccine. Mother education, mothers’ perception to accessibility of vaccines, mothers’ knowledge to vaccine schedule of their site, place of delivery and living altitude were independent predictors of children immunization status.

**Conclusion:**

Expanded program of immunization (EPI) coverage at Arba Minch town and Arba Minch Zuria district is better than the national immunization coverage but still below the goal. Educating mother, promoting institution delivery could help to maintain and enhance current immunization coverage. More emphasis should be given to the highland areas of the area.

## Background

Worldwide about 29,000 children under the age of five die every day, mainly from preventable causes. Every year around 8 million children in developing countries die before they reach their fifth birthday; many during the first year of life. An Ethiopian child is 30 times more likely to die by his or her fifth birthday than a child in Western Europe [1,2].

Ethiopia placed sixth among the countries of the world in terms of child death as about 472,000 Ethiopian children die each year before their fifth birthday. Most of these deaths occur from few preventable and curable diseases and conditions [3].

During 2010–2011, a total of 9,756 measles cases were reported in Ethiopia; more than three fourth of it occurred among children age <5 years. Cases occurred predominantly in unvaccinated persons. Keffa was one of the zones experienced a large measles outbreak between October 2011 and February 2012 where over 3,000 measles cases were confirmed and thousands of unimmunized children registered in the Zone [4,5].

Immunization currently averts an estimated 2 to 3 million deaths every year. However, the proportion of the world’s children who receive recommended vaccines has remained steady for the past few years [4].

According to guidelines developed by the World Health Organization (WHO), children are considered fully vaccinated when they have received a vaccination against tuberculosis Bacillus Calmette Guerin (BCG), three doses each of diphtheria, pertussis and tetanus toxoids (DPT),three doses of polio vaccines, and a measles vaccination by the age of 12 months. Since Ethiopia included Haemophilus influenza type B (HiB) and hepatitis B(HepB) as of 2007; pentavalent vaccine (DPT plus Hep B and Hib) has replaced the previous DPT vaccine [6,7].

World Health organization reports indicated by the year 2011 worldwide coverage of three doses of DTP, one dose of measles and three doses of polio vaccine were 83%, 84% and 84% respectively. However, more than one-fifth of the world’s children, especially those in low-income countries, were still not fully vaccinated at 12 months of age and remained at risk for vaccine-preventable morbidity and mortality [4,8].

Complete immunization coverage in Kenya by the year 2011 was 76.6% with 99.5% BCG and 77.4% measles coverage. Identified predictors were number of children within the family, place of birth of the child, and opinion on the health immunization services offered [9]. In 2005 Indian complete status immunization was 44% almost similar with Pakistan and factors identified were mother education, mother religion and child birth order [10,11].

In Ethiopia, the vaccination policy calls for BCG vaccine given at birth or at first clinical contact, three doses of pentavalent (DPT-HepB-Hib) vaccine and the newly added pneumococcal conjugate vaccine (PCV) vaccine given at approximately 4, 8, and 12 weeks of age, four doses of oral polio vaccine given approximately at 0-2, 4, 8, and 12 weeks of age, and measles vaccine given at or soon after reaching 9 months of age [6,12]. All these vaccines are recommended to be given within one year of a child age.

Ethiopian central statistics agency in its 2011 demographic and health survey found that 24% of Ethiopian children 12–23 months age have received all recommended vaccines; one dose each of BCG and measles, and three doses each of DPT and polio (excluding polio zero vaccine given at birth). The finding of this survey showed improved immunization coverage as compared with similar survey in 2005 which revealed that only 20% of children 12–23 at the time. The 2011 survey also reported fifteen percent of children did not receive any of the recommended vaccines. Vaccination coverage is more than double in urban areas than in rural areas of the country (48% versus 20%) [13,14]. In contrast with this national survey other studies; a 2006 survey reported 49.9% in nationwide, a 2008 survey in Wonago district 41.7% and in 2011 at Ambo district 36% of the children were fully vaccinated [2,7,15].

The study in Wonago district also identified knowledge of the mothers about child immunization, monthly family income, postponing child immunization and perceived health institution support were the predictors of defaulting from completion of child immunization [2].

In 2011 Belachew and his colleague reported 23.7% children of Ambo district were no vaccinated at all. They also identified factors affecting complete immunization as sex of child, antenatal care follow-up, being born in the health facility, and mothers’ knowledge about the age children start and complete vaccination [7].

Ethiopian demographic and health survey (EDHS) 2011 also calculated coverage for specific vaccines, 66 percent of children received the BCG vaccine, and 56 percent had received the measles vaccine. A relatively high percentage of children received the first DPT dose (64%). However, only 37% went on to receive the third dose of DPT. More than eight children of every ten (82%) received the first dose of polio, but only 44% received the third dose [13].

According to guidelines developed by the WHO and shared by Ethiopia, a child is considered fully immunized when he/she has received one dose of BCG, three doses of DPT (since Ethiopia included HiB and HepB since 2007; pentavalent vaccine (DPT + HepB_Hib) has replaced the previous DPT vaccine), three doses polio vaccines excluding polio zero given at birth and a measles vaccination by the age of 12 months. A child is considered as partially immunized if received any vaccines but missed at least one dose of one BCG, three doses of pentavalent, three doses of oral polio vaccine (OPV) and a measles vaccine. A child is considered as not immunized if he/she does not receive any dose of vaccines [12,16].

Countries including Ethiopia are aiming to achieve vaccination coverage of ≥90% nationally and ≥80% in every district by the year 2020. Reports indicated percentage of children who are fully vaccinated remains far below the goal set in the fourth Ethiopian health sector development plan (HSDP IV) [4,12]. Frequent measles outbreak and high child morbidity and mortality rate in the country are among the consequences of low immunization coverage. Many children in Ethiopia do not get the benefits of immunization [3,17].

The Ethiopian government is striving to reduce child mortality rate. One of the means is by increasing immunization coverage with several being implemented strategies. The Ethiopian ministry of health (EMoH) has newly incorporated PCV vaccine in its EPI program as of 2011. One year earlier to the period of current study when study subjects were expected to be immunized Ethiopian Ministry of Health with its partners had conducting survey in the current study area. During the survey period vaccine promotions were always held in every vaccination day. Generally with the above mentioned reasons and others more, vaccine coverage was expected to be improved.

To have evidence based immunization plan and vaccination and to increase the immunization coverage, immunization coverage and its predictors has to be identified through community based studies. This study was required to be conducted because there was no such study conducted in the area. Vaccination coverage reports from different health institutions of the area were different from other published study reports. But no other similar studies were done to measure level of immunization coverage and predictors of immunization in current study area.

Even though some community based studies are available with different findings conducted in other parts of the country, there was no other similar study conducted in current study area. The objective of this study is to measure EPI coverage and to identify the predictors of immunization among children between 12–23 months of age in Arba Minch Town and Arba Minch Zuria District, Southern Ethiopia.

## Methods

Community based cross-sectional study has been conducted in Arba Minch town and Arba Minch Zuria district, Gamo Gofa Zone, Southern Ethiopia in March 2013. In Arba Minch town one referral hospital and two health center are serving for 40 districts of the town and Arba Minch district and other surrounding districts [18].

The study population was children age 12 to 23 completed months in selected 15 districts of Arba Minch town and Arba Minch Zuria district. Ethiopian national census of 2007 report shows 2.7% of the total populations were children of age 12 – 23 months [13].

Samples of 630 children aged 12–23 months and their caretakers/mothers were involved in the study. Calculation was done using a single proportion population formula with a 95% confidence level, 5% margin of error and 24% immunization coverage rate in the study region (Southern Nations, Nationalities and Peoples Regional State (SNNPR) fully immunized percentage). Ten percent non-response rate and design effect of 2 were considered [13].

To select a study unit the 2005 WHO EPI cluster sampling method was used. Fifteen districts (5 from Arba Minch town and 10 from Arba Minch Zuria District) from 40 districts have been selected randomly and each district was managed as a cluster. In the selected districts, first enumeration was done to identify all eligible children. Determined samples (630) were proportionally distributed to each cluster (probability proportional to size (PPS))based on number of eligible children in each cluster after enumeration of selected clusters. Using house code given at the time of enumeration eligible study children were selected randomly [6].

Ethical clearance was obtained from the Ethical Review Committee of Arba Minch University. Before data collection, oral consent was obtained from the respondents.

Data were collected with semi-structured pretested Amharic version questionnaire. The data collection questionnaire had been adopted from world health organization (WHO) and Demographic health survey of Ethiopia (DHS) [6,11]. The adopted English version questionnaire was translated in to Amharic and back to English to assure consistency. To ensure data quality, pre-test was done in one district which was not included in the main study.

Thirty grade 10 completed data collectors five nurse supervisors were trained and recruited. Data were collected from mothers or caretakers by interview. Only one mother/care taker was interviewed for each index child. Information about children immunizations were collected from children immunization cards. If immunization cards were lost then the maternal/caretakers report of immunizations were recorded. Presence of BCG scar was observed and recorded.

Data entry was done by EpiInfo and exported to SPSS version 16 statistical software. Data cleaning, coding and analyzed were performed using the SPSS statistical software. Dependent variables were dichotomized into fully immunized and not fully immunized (partially immunized and not immunized children) sharing the definition given by WHO based on the information from children immunization card. To explain the study population in relation to relevant variables, frequencies and summery statistics were used. Associations between dependent and independent variables were assessed and presented using three consecutive logistic regression models. In the first model, the effects of socio-demographic variables were assessed. The effects of vaccine accessibility were assessed in the second model. From two models, variables which had significant association with dependent variable (P < 0.05) were entered to the final third regression model.

## Results

### Socio-demographic characteristics

This study has measured the EPI coverage within 630 children age 12 – 23 months residing in 15 districts of Arba Minch Town and Arba Minch Zuria districts. Nearly half, 48.4% of the study participant children were females. The mean and median age of study children were 17.4 and 17 months respectively. More than seventy percent of the children were born out of health institutions most of them by none health professionals; traditional birth attendants, family members, neighbors or with no attendant and some by local health extension workers. Majority (88.3%) of children were living with their both parents at the time of study Table 1.

**Table 1:** Socio-demographic characteristics of parents and children age 12 – 23 months of Arba Minch town and Arba Minch ZuriaDistrict, Southern Ethiopia, 2013; (N = 360)

**Variable**		**Category**	**Frequency (%)**
Index child	Sex of index child	Male	325 (51.6)
		Female	305 (48.4)
	Birth place	Home	446 (70.8)
		Health institution	184 (29.2)
	Birth attendant	Mother alone	68 (10.8)
		Family member	177 (28.1)
		Neighbor	70 (11.1)
		Traditional birth attendant	88 (14)
		Health professional	116 (34.4)
		Other	11 (7.1)
	Living condition	Both parents	556 (88.3)
		Mother only	52 (8.3)
		Grand parents	22 (3.5)
Mother	Ever ANC visit for index child	Yes	569 (90.3)
		No	61 (9.7)
	Residence	Urban	178 (28.3)
		Rural	452 (71.7)
	TT vaccine taken	Yes	459 (72.9)
		No	171 (27.1)
	Mother religion	Protestant	423 (67.1)
		Orthodox	183 (29.0)
		Muslim	4 (0.6)
		Other	20 (3.2)
	Mother marital status	Married	582 (92.4)
		Not married	30 ( 4.8)
		Divorced	9 (1.4)
		Widowed	9 (1.4)
	Mother education status	No education	262 (41.6)
		Primary school	252 (40.0)
		High school	116 (18.4)
	Mother occupation	House wife	510 (81.0)
		Daily laborer	31 (4.9)
		Private worker	49 (7.8)
		Government employed	21 (3.3)
		Other	19 (3.0)
Father	Father education	No education	159 (25.2)
		Primary school	293 (46.5)
		High school	178 (28.3)

The mean and median ages of study children’s mother were 25.3 and 25 years respectively. Majority of parents were residing in rural and fellow of protestant religion. Majority of mothers were house wives (81%) and most (92.4%) were married. More than half (58.4%) of mothers had formal education. Most of the mothers (90.3%) had at least one antenatal care (ANC) visit while pregnant for the study child. More than quarter (27.1%) of the mothers had never immunized against tetanus Table 1.

### Accessibility to vaccine

The mothers of study children were asked about their knowledge, perception and practice to vaccine and accessibility of vaccine to their residence. Most of the participants responded there were accessible vaccination site to their residing area and knew vaccine schedule of the site 91.6%, 82.9% respectively. For more than half (56.0%) of mothers the travel time to vaccine site were less than 15 minutes. Around half (51.7%) of the participants were using health post for their children immunization. The waiting time for 38.1% was less than 15 minutes and for 26.7% the waiting time at the vaccination site was more than an hour. Most of participants responded that their vaccination site had never cancel schedule and their children never returned without getting vaccine 97.2% and 87.2% correspondingly Table 2.

**Table 2:** Vaccine accessibility and mothers’ perception to vaccine accessibility, Arba Minch Town and Arba Minch Zuria District, Southern Ethiopia, 2013, (N = 630)

**Variable**	**Category**	**Frequency (%)**
Is there accessible vaccination site to yourresiding area?	Yes	577 (91.6)
	No	53 (8.4)
Vaccine site to the area	Hospital	17 (2.7)
	Health center	166 (26.3)
	Health post	326 (51.7)
	Local vaccine site	118 (18.8)
	Other	3 (0.5)
Travel time to Nearest Vaccine site	< 15 min	353 (56.0)
	15 – 30 min	177 (28.1)
	30 – 60 min	82 (13)
	>one hour	18 (2.9)
Waiting time	< 15 min	240 (38.1)
	15 – 30 min	120 (19.0)
	30 – 60 min	101 (16.0)
	>one hour	169 (26.8)
Know local Vaccine site schedule	Yes	522 (82.9)
	No	108 (17.1)
Any cancelation of vaccine schedule	Yes	52 (2.8)
	No	578 (97.2)
Ever return without getting vaccine	Yes	106 (16.8)
	No	524 (83.2)

### Children immunization status

**Figure 1:**
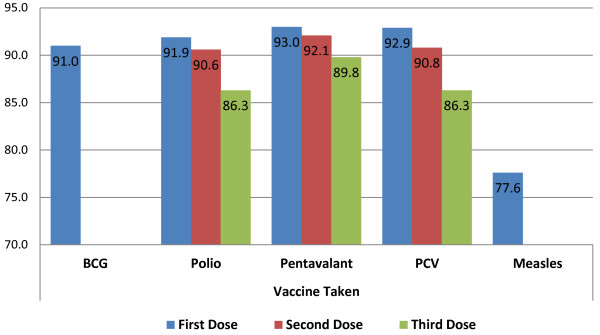
Vaccine taken by study children age 12 – 23 months, Arba Minch Town and Arba Minch Zuria District, Southern Ethiopia, 2013 (N = 630).

**Figure 2:**
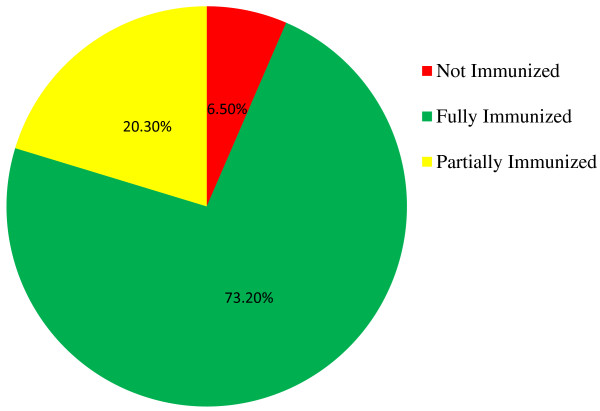
Immunization status of children age 12 – 23 months, Arba Minch Town and Arba Minch Zuria District, Southern Ethiopia, 2013, (N = 630).

Information about the immunization status of study children were collected from both the mother/guardian and vaccine card. Most study children had taken all vaccines given in the country including the newly incorporated three dose PCV (pneumococcal conjugate vaccine) vaccine. As depicted in the Figure 1 relatively less percentages of children had taken measles (77.6%) as compared with 91.0% of children were vaccinated for BCG. As shown in the figure below corresponding vaccination reduction has been recorded from first to second and third doses of Polio, Pentavalant and PCV vaccines. Even though 93.0%, 92.9% and 91.9% of children had started Pentavalant, PCV and Polio vaccines only 89.8%, 86.3% and 86.3% had completed the recommended third doses of each vaccine respectively (Figure 2).

Sharing the definitions given by WHO nearly three fourth (73.2%) of children in Arba Minch Town and Arba Minch Zuria district are fully immunized. As depicted in Figure 2 the rest 20.3% and 6.5% are partially immunized and not immunized/vaccinated respectively (Figure 2).

### Factors contributing to immunization status

Predictors for immunization of children have been assessed by dichotomizing dependent variable into fully immunized and not fully immunized (partially immunized and not immunized children) depending on the information from children’s immunization cards. Among the socio-demographic variables computed in the first model place of delivery, mother’s education status and living area are found to be the contributing factors for children’s immunization status. Nearly two third (67.4%) of children delivered at home were found to be fully immunized as compared with 87.0% of children who had been delivered in health institution AOR (95% CI) 0.41(0.17, 0.98). The other identified factor in the first model is mothers’ educational status. Children from educated mothers had better chance to be fully immunized than children from not educated mother AOR (95% CI) 2.22(1.31, 3.76). Almost half (51.4%) of children in highland area were fully immunized as compared with (81.5%) of children reside in lowland area of study site AOR (95% CI) 0.48(0.29, 0.80).

Other variables; sex of children, birth attendant, ever have ANC visit for index children, residence, mother age, mother occupation, mother religion and father education are found not to have statistical significant impact on children’s immunization status among variables computed in first logistic regression model (Table 3).

**Table 3:** Socio-demographic factors and children immunization status of Arba Minch Town and Arba Minch Zuria District, Southern Ethiopia, 2013 (N = 630)

**Variable**	**Category**	**Fully immunized**		**Model I**	
		**No**	**Yes**	**COR (95% OR)**	**AOR (95% OR)**
		**N (%) 169 (26.8)**	**N (%) 461 (73.2)**		
Sex of index child	Female	78 (25.6)	227 (74.4)	1.13 (0.79, 1.61)	NI
	Male	91 (28.0)	234 (72.0)	1	
Place of delivery	Health institution*	24 (13.0)	160 (87.0)	1	
	Home	145 (32.5)	301 (67.5)	0.31 (0.19, 0.49)	0.41 (0.17, 0.98)
Birth attendant of index child	Health professional**	34 (15.7)	182 (84.3)	1	
	Not health professional	135 (32.6)	279 (67.4)	0.38 (0.35, 0.58)	1.52 (0.69, 3.37)
Ever ANC visit for index child	Yes	145 (25.5)	424 (74.5)	1.89 (1.09, 3.27)	0.91 (0.49, 1.67)
	No	24 (39.3)	37 (60.7)	1	1
Residence	Rural	142 (31.4)	310 (68.6)	1	
	Urban	27 (15.2)	151 (84.8)	2.56 (1.65, 4.03)	1.19 (0.70, 2.02)
Mother age	<24 years	55 (22.3)	192 (77.7)	1	1
	25 – 34 years	101(29.9)	237 (70.1)	0.67 (0.46, 0.98)	1.05 (0.68, 1.61)
	>35 years	13 (28.9)	32 (71.1)	0.70 (0.34, 1.43)	1.58 (0.72, 3.49)
Mother religion	Protestant	113 (26.7)	310 (73.3)	1	NI
	Orthodox	47 (25.7)	131 (74.3)	1.05 (0.71, 1.56)	
	Muslim	1 (25.0)	3 (75.0)	1.09 (0.11, 10.62)	
	Other	8 (40.0)	12 (60.0)	0.54 (0.21, 1.37)	
Mother marital status	Married	159 (27.3)	423 (72.7)	1	NI
	Never married	6 (20.0)	24 (80.0)	1.50 (0.60, 3.74)	
	Divorced	1 (11.1)	8 (88.9)	3.00 (0.37, 24.23)	
	Widowed	3 (33.3)	6 (66.7)	0.75 (0.18, 3.04)	
Mother education status	No education	112 (42.7)	150 (57.3)	1	1
	Primary school	41 (16.2)	211 (83.8)	3.84 (2.53, 5.81)	2.22 (1.31, 3.76)
	High school and above	16 (13.8)	100 (86.2)	4.66 (2.60, 8.34)	2.19 (0.97, 4,94)
Father education status	No education	69 (43.4)	90 (56.6)	1	1
	Primary school	74 (25.2)	219 (74.8)	2.26 (1.50, 3.41)	1.32 (0.83, 2.11)
	High school and above	26 (14.6)	152 (85.4)	4.48 (2.66, 7.54	1.19 (0.70, 2.02)
Living area	Low land	84 (18.5)	371 (81.5)	1	1
	High land	85 (48.6)	90 (51.4)	0.24 (0.16, 0.35)	0.48 (0.29, 0.80)

*To the context of this study *health institution* means health post, clinic (both governmental and none governmental), health center and hospital.

**Health professionals mean health extension workers, midwives, nurses, health officers and physicians.

More than three fourth (76.1%) of children whose mothers perceived vaccine site was accessible to their residence area were fully immunized; whereas only 41.7% of children whose mother perceived in reverse were fully immunized AOR (95% CI) 4.54(2.34, 8.77). Mothers knowledge to local vaccine site schedule is the other identified factor contributing to children’s immunization status; 59.3% of children whose mothers did not knew local vaccine site schedule were fully immunized as compared with 76.1% of children whose mothers knew vaccine schedule of their site were fully immunized AOR (95% CI) 0.43(0.27, 0.70) (Table 4).

**Table 4:** Vaccine accessibility and children immunization status of Arba Minch Town and Arba Minch Zuria District,Southern Ethiopia, 2013 (N = 630)

**Variable**	**Category**	**Fully immunized**		**Model II**	
		**No**	**Yes**	**COR (95% OR)**	**AOR (95% OR)**
		**N (%) 169 (26.8)**	**N (%) 461 (73.2)**		
Is there accessible vaccination site to your area?	No	31 (58.3)	22 (41.7)	1	
	Yes	138 (23.9)	439 (76.1)	4.48 (2.51, 7.99)	4.54 (2.34, 8,77)
Know immunization schedule of the area	Yes	125 (23.9)	397 (76.1)	1	1
	No	44 (40.7)	64 (59.3)	0.45 (0.29, 0.70)	0.43 (0.27, 0.70)
Nearest vaccine site	Health post	103 (31.6)	223 (68.4)	1	1
	Outreach	28 (23.6)	90 (76.3)	1.48 (0.91, 2.40)	1.89 (1.07, 3.33)
	Health center	34 (20.5)	132 (79.5)	1.79 (1.51, 2.79)	1.73 (1.08, 2.79)
	Hospital	2 (11.8)	15 (88.2)	3.46 (0.77, 5.42)	4.45 (0.95, 20.82)
	Other	2 (66.6)	1 (33.3	0.23 (0.02, 2.57)	0.72 (0.04, 1.02)
Travel time to nearest vaccine site	< 15 min	85 (24.1)	268 (75.9)	1	1
	15 – 30 min	47 (26.6)	130 (73.4)	0.87 (0.58, 1.32)	0.82 (0.53, 1.26)
	30 – 60 min	24 (29.3)	58 (70.7)	0.76 (0.44, 1.30)	0.94 (0.52, 1.67)
	>one hour	13 (72.2)	5 (27.8)	0.12 (0.04, 0.35)	0.24 (0.02, 0.26)
Vaccine schedule ever postponed	Yes	15 (28.8)	37 (71.2)	1	NI
	No	154 (26.6)	424 73.4)	1.11 (0.59, 2.09)	
Ever return without getting vaccine	Yes	27 (25.5)	79 (74.5)	1	NI
	No	142 (27.1)	382 (72.9)	0.91 (0.57, 1.48)	

Outreach scheduled vaccine sites and health centers had better success to give full immunization to the children of their catchment area as compared with health post AOR (95% CI) 1.89(1.07, 3.33). Only quarter of children were not fully immunized if travel time to vaccine site was less than 15 minutes in contrary nearly three fourth, 72.2% of children were not fully immunized if the travel time was above one hour AOR (95% CI) 0.24(0.02, 0.26) (Table 4).

## Discussion

This cross-sectional community based study was conducted in March 2013 at Arba Minch town and Arba Minch Zuria district to determine EPI (Expanded Program of Immunization) coverage at the area. This study has revealed the immunization coverage at the area is better than the national and regional coverage. Nearly three fourth (73.2%) of children in the study area were fully immunized; 90.0% and 77.6% had taken one doses of each BCG and Measles, 89.8% and 86.3% had taken three doses of each Pentavalant and Polio and vaccines respectively. Only 6.5% did not take any vaccine at all. The percentage of not immunized children is significantly lower than the figures of nationwide (15%) and Ambo district (23.7%) [7,14].

Even though this percentage (73.2%) of fully immunized children is lower than EDHS 2011 report of children in Addis Ababa (78.9%) and peri-urban area of Kenya (76.6%), it is higher than any other national reports; 2011 nationwide (Ethiopia) EPI coverage 24.3% and 2005 Indian immunization coverage 44.0% [9,11,14]. A community based study in 2011 in Ambo district had found that fully immunized children in the area were 27.7% and other study done in SNNPR Wonago district found it was 41.7% [2,7]. Other Ethiopian nationwide survey in 2006 also found that half (49.9%) of children age 12 -23 were fully immunized [15].

Even though there is no matched control data to compare current finding, there is one concrete reason for better EPI coverage at the area during the study period. The Ethiopian ministry of health (EMoH) has newly incorporated PCV vaccine in its EPI program since November 2011. Since this vaccine was new to the country and has new packaging system (preservative free two doses with in a vial; the standard and other countries using is single dose within a vial), EMoH with its partners were conducting survey in selected sites of the country. Arba Minch Town and Zuria districts was among these sites. During the PCV survey period all eligible children (age > 1 year) were being registered and mothers were convinced to took their children for vaccine. Vaccine promotions were always held in every vaccination day. All children who did not come or missed their schedule were being traced. There were better documentations at the time of the survey. This was the period that current study participants were expected to be vaccinated. Hence, it is not surprising if more children had received recommended vaccines at this period.

The Ethiopian government is striving to reduce child mortality rate and one of the means is by increasing immunization coverage with several being implemented strategies. Generally with the above mentioned reasons vaccine coverage in the current study has been recorded better than any other previous study findings.

All variables showed association in the first and second logistic regression models have been entered in the last/third logistic regression model. Even though nearest vaccination site and travel time to vaccination site had shown statistical significant effect on children’s immunization status, they could not maintain their statistical significancy while adjusted with other variables in third model; others are still the factors affecting the dependent variable (Table 5).

**Table 5:** Factors contributing to children age 12 – 23 months immunization status of Arba Minch Town and Arba Minch Zuria District, Southern Ethiopia, 2013 (N = 630)

**Variable**	**Category**	**Fully immunized**		**Model III**	
		**No**	**Yes**	**COR (95% OR)**	**AOR (95% OR)**
		**N (%) 169 (26.8)**	**N (%) 461 (73.2)**		
Is there accessible vaccination site to your area?	No	31 (58.3)	22 (41.7)	1	1
	Yes	138 (23.9)	439 (76.1)	4.48 (2.51, 7.99)	3.07 (1.52, 6.18)
Nearest Vaccine site	Health Post	103 (31.6)	223 (68.4)	1	1
	Health Center	34 (20.5)	132 (79.5)	1.79 (1.51, 2.79)	0.55 (0.30, 1.01)
	Outreach	28 (23.6)	90 (76.3)	1.48 (0.91, 2.40)	1.16 (0.61, 2.20)
	Hospital	2 (11.8)	15 (88.2)	3.46 (0.77, 15.42)	1.60 (0.30, 8.38)
	Other	2 (66.6)	1 (33.3	0.23 (0.02, 2.57)	0.18 (0.01, 3.13)
Travel time to Nearest Vaccine site	< 15 min	85 (24.1)	268 (75.9)	1	1
	15 – 30 min	47 (26.6)	130 (73.4)	0.87 (0.58, 1.32)	1.12 (0.70, 1.80)
	30 – 60 min	24 (29.3)	58 (70.7)	0.76 (0.44, 1.30)	1.83 (0.98, 3.42)
	>one hour	13 (72.2)	5 (27.8)	0.12 (0.04, 0.35)	0.60 (0.18, 1.96)
Know immunization schedule of the site	Yes	125 (23.9)	397 (76.1)	1	1
	No	44 (40.7)	64 (59.3)	0.45 (0.29, 0.70)	0.40 (0.24, 0.68)
Mother Education Status	No Education	112 (42.7)	150 (57.3)	1	1
	Primary School	41 (16.2)	211 (83.8)	3.84 (2.53, 5.81)	2.03 (1.21, 3.38)
	High school and above	16 (13.8)	100 (86.2)	4.66 (2.60, 8.34)	2.27 (1.12, 4.60)
Place of Delivery	Health Institution	24 (13.0)	160 (87.0)	1	1
	Home	145 (32.5)	301 (67.5)	0.31 (0.19, 0.49)	0.47 (0.26, 0.85)
Living Area	Low Land	84 (18.5)	371 (81.5)	1	1
	High Land	85 (48.6)	90 (51.4)	0.24 (0.16, 0.35)	0.33 (0.18, 0.60)

The odds of children from mothers who perceived vaccine site was accessible to their residing area were 3 times more likely to be fully immunized than children from mothers who perceived in reverse, [AOR (95% CI) = 3.07(1.52, 6.18)]. Children whose mothers did not knew the vaccine schedule of their site had less chance to be fully immunized, [AOR (95% CI = 0.40(0.24(0.68)]. This finding is in line with the previous case-control study in Wonago district [2] (Table 5).

Ethiopian Federal Ministry of Health is striving to improve vaccine accessibility; all rural kebles have one health post giving all vaccines and all rural villages have at least once every month outreach vaccine schedule. Even though one hospital and two health centers in study area are giving vaccine in all working days and hours, all other health centers, health posts and outreach vaccine sites are giving vaccines only on monthly schedule. Therefore if children missed the vaccination day of their site, they must either wait to the next month’s vaccine day or visit other sites. When mothers perceived vaccination site was not accessible and they did not knew vaccine schedule of their site, they might not take their children for vaccination. Many previous studies identified that mothers’ education significantly affects children’s immunization status [2,7,11,15]. The current study also reasserted that the better mothers were educated the more their children had been fully immunized, [AOR (95% CI) = 2.27(1.12, 4.60)].

Only 13% of children who had been delivered in health institution were not fully immunized as compared with 32.5% of children delivered home. The study revealed that the odds of children who had been born homes were less to be fully immunized than children who had been born in health institutions, [AOR (95% CI) = 0.47(0.26, 0.85)] which is in line with previous study in Ambo district [7].

Ethiopian EPI schedule recommends Polio zero and BCG to be given at birth. When a child had been delivered in health institution he/she could start taking OPV zero and BCG vaccines and mothers could be informed about vaccines and when and where to return for next vaccines. This might be one of the possible reasons children born in health institution were better immunized.

The final logistic regression model also showed statistical significant association between living altitude and children’s immunization status. The odds of children in highland area to be fully immunized were 0.33 times as compared with children residing in lowland areas of the study site [AOR 95% CI) = 0.33(0.18, 0.60). This could be due to all kebles in highland were rural and the transportations to these sites were difficult, most roads were season dependent; not accessible in rainy season. Even though each districts of the highland areas had health post and at least two health extension workers, there might be problems in vaccines supply to each site on time keeping the cold chain, supervisions were not as tight as the lowland, and houses within kebles were too sparse and geographical placement is totally impossible for vehicle and difficult for walk (Table 5).

## Conclusion

EPI coverage at Arba Minch town and Arba Minch Zuria district is better than the national immunization coverage. Mothers’ perception to accessibility of vaccine site and knowledge to vaccine schedule are among the predictor factors for children’s immunization status. Children’s immunization coverage in highland parts of Arba Minch Zuria district is significantly lower than children residing lowland area of the study site.

## Competing interests

The authors declare that they have no competing interests.

## Authors’ contributions

WA has involved in the conception, design, analysis, data interpretation and report writing. WT, BM, MT and GA have involved in the design, analysis and report writing. All authors read and approved the final manuscript.

## Pre-publication history

The pre-publication history for this paper can be accessed here:

http://www.biomedcentral.com/1471-2458/14/464/prepub
